# Adolescent hope and optimism: A scoping review of measures and their psychometric properties

**DOI:** 10.4102/ajopa.v5i0.137

**Published:** 2023-09-26

**Authors:** Esther J. Chongwo, Ezra K. Too, Adam A. Mabrouk, Amina Abubakar

**Affiliations:** 1 Institute for Human Development, Aga Khan University, Nairobi, Kenya

**Keywords:** optimism, hope measures, adolescents, scoping review, psychometric properties

## Abstract

There is much research examining adolescent hope and optimism, but there is limited information on the available measures, particularly the most frequently used measures and their psychometric properties. We conducted a scoping review to: (1) identify measures of hope and optimism for use among adolescents; (2) determine the most frequently used measures of adolescent hope and optimism; (3) document the psychometric properties of the identified measures. We searched five bibliographic databases and Open Grey for relevant articles published from database inception to 03 May 2023. The search included the key terms ‘adolescents’, ‘optimism’, ‘hope’ and ‘measures’. We targeted adolescents aged 10–19 years without country or gender restrictions. We identified 86 measures of optimism and 64 measures of hope for use among adolescents from 803 eligible studies. Most of the included studies (*n* = 341, 42.5%) originated from North America. We identified 22 most frequently used measures used across 603 (75.1%) of the included studies, with 509 (84.4%) of them providing their psychometric properties. The reported validity of the tools included face, content, construct, criterion, convergent, discriminant, predictive validity and measurement invariance. There are several measures of adolescent hope and optimism. The most frequently used measures of these constructs demonstrated sound psychometric properties, especially reliability. However, most of the evidence originates from high-income countries. There is a need for development, cross-cultural adaptation and validation of these tools to other settings.

**Contribution:**

Hope and optimism are character strengths that have been consistently linked to positive health outcomes in adolescents. Based on increasing research on adolescent hope and optimism, there have been measures developed to assess these constructs but there is no study summarising the available measures, particularly regarding the most frequently used measures and their reliability and validity across contexts. This study aimed at filling this gap. Information on this will be useful to various stakeholders to make evidence-informed choice on selection of the most relevant instrument for use in adolescents in their contexts.

## Introduction

Adolescence is a critical stage characterised by socio-emotional, psychological, physical and intellectual growth and development (Bowers et al., 2010; Lerner et al., 2005; Snyder et al., 1997). It is thought that during this period, individuals are malleable and are capable of developing habits, virtues, skills, competencies and knowledge that will be useful in later life (Allemand et al., 2015). As such, adaptive or maladaptive experiences can also occur during this period; hence, there is a need to view adolescents as resources to be developed and not problems to be managed (Roth & Brooks-Gunn, 2003). Therefore, this is a unique stage for positively moulding an individual’s character to ensure they thrive and flourish in the society. Character development, one of the 6Cs of positive youth development, is thought to involve acquiring positive mental and behavioural attributes through person and context interactions (Lerner et al., 2012).

Adolescent character development has been linked to various health benefits, including improved self-esteem (Fenton et al., 2010) and healthy behaviours such as nutrition, exercise and sexual health, among others (Catalano et al., 2004; Hoyt et al., 2012). Hope and optimism are character strengths that have been consistently linked to positive health outcomes in adolescents, including mental and physical health (Häggström Westberg et al., 2019). Snyder et al. (1997) defined hope as belief in one’s abilities to seek for opportunities (pathway) in order to attain their goals (agency) (Snyder et al., 1997). Agency refers to an individual’s belief in their capacity to initiate, control and influence actions towards attainment of desired goals, while pathway refers to the perception of having viable strategies or resources that can facilitate one’s progress towards achieving the desired goals (Snyder et al., 1997). In essence, hope involves having a positive attitude for the future combined with the belief that one has the agency to actively pursue goals and the pathway to attain them (Snyder et al., 1997). In contrast, optimism has been defined as general positive expectations about future outcomes (Scheier & Carver, 1992). Individuals with a sense of agency are more likely to hold optimistic beliefs and feel confident in their ability to navigate challenges.

Hope and optimism are closely related constructs. In the literature, there is a blurred distinction between hope and optimism, and these two concepts have been frequently used interchangeably (Gillham & Reivich, 2004). These concepts share a common theme of belief in the future/future orientation (Catalano et al., 2004; Sun & Shek, 2012). However, other studies have argued that these concepts could be distinct but overlapping constructs (Bryant & Cvengros, 2004; Magaletta & Oliver, 1999; Snyder, 2000). Despite their close relationship, optimism involves a generalised expectation of positive outcomes and the belief that adversity is less likely to occur (Scheier & Carver, 1985). In contrast, hope is more goal-oriented and specific, involving the setting of goals, identification of pathways to achieve them, and maintaining motivation and agency in pursuing those goals (Snyder, 2000). Overall, both constructs have consistently been seen as valued positive youth development strengths that enhance positive outcomes (McCoy & Bowen, 2015; Sun & Shek, 2012).

Optimism in adolescents has been reported to have protective effects against mental health problems (Ames et al., 2015), such as anxiety (Dooley et al., 2015), substance abuse (Ansari et al., 2019), depressive disorders (Ames et al., 2015), and suicide (Tanner et al., 2014). Other documented benefits include enhanced social relations, greater academic achievement, better problem-solving skills and positive coping strategies (Carver & Scheier, 2014). Similarly, hope has been documented to enhance youth’s life satisfaction, improve self-esteem (Park et al., 2004), predict academic achievement (Ciarrochi et al., 2007) and psychological wellbeing (Valle et al., 2006). Additionally, hopeful children have enhanced coping strategies (Hellman & Gwinn, 2017), and have improved problem-solving skills (Pedrotti et al., 2008).

Based on increasing research on adolescent hope and optimism, there have been measures developed to assess these constructs. Some of these measures include the Life Orientation Test (LOT) (Scheier & Carver, 1985), the Children’s Hope Scale (CHS) (Snyder et al., 1997), and the Youth Life Orientation Test (YLOT) (Ey et al., 2005), among others.

There is a growing interest in adolescent hope and optimism (Schiavon et al., 2017; Seligman & Csikszentmihalyi, 2000; TenHouten, 2023), although little is known concerning their available measures, particularly regarding the most frequently used measures and their psychometric properties. Where it has been documented in the literature, the report has been limited to certain regions. For instance, a recent review on available measures of hope and optimism only focused on studies conducted in Western countries and China, and lacked information on the psychometric properties of the measures (Leung et al., 2017). Given the importance of hope and optimism on various developmental and health outcomes, there is a need to summarise data on the tools and synthesise the evidence on their reliability and validity.

To fill this research gap, we conducted a scoping review of existing measures of hope and optimism, and their psychometric robustness. Scoping reviews are usually conducted to explore the scope of literature, and to map out and provide a summary of existing evidence to inform future research (Tricco et al., 2016). Information on this will be useful to researchers, practitioners and policymakers to make evidence-based choices on selection of the most relevant instrument for use for optimism in adolescents in their contexts. This study therefore aims to:

- Identify the measures of hope and optimism available for use among adolescents.
- Determine the most frequently used measures of adolescent hope and optimism.
- Document the psychometric properties of the identified measures of hope and optimism in adolescents including their reliability utility and validity across-cultural contexts.

## Methods

This study was conducted following the scoping review framework by Arksey & O’Malley (2005), which entails the following steps: (1) identifying research question(s); (2) identifying relevant studies; (3) study selection; (4) extracting and charting the data; (5) collating, summarising, and reporting the results.

### Identification of research question(s)

This study aimed at addressing the following research questions:

- Which are the measures of hope and optimism available for use among adolescents?
- Which are the most frequently used measures of adolescent hope and optimism?
- What are the psychometric properties of the identified measures of hope and optimism in adolescents including their utility and validity across-cultural contexts?

### Identification of relevant studies

We conducted a search in five bibliographic databases, including PubMed, PsycINFO, Embase, CINAHL and Web of Science, for articles published from database inception to 03 May 2023 (the day of the last search). We also searched for relevant articles from the grey literature, specifically in Open Grey. Additionally, we checked the reference lists of the included papers for additional relevant literature. We included the keywords ‘adolescents’, ‘optimism’ ‘hope’ and ‘measures’ combined by the Boolean operator ‘and’. The respective synonyms for these keywords were joined using the ‘or’ Boolean operator. The search strategy was adapted to fit the specifications of the different databases.

Online Appendix 1 shows the search terms used.

### Study selection

**FIGURE 1:**
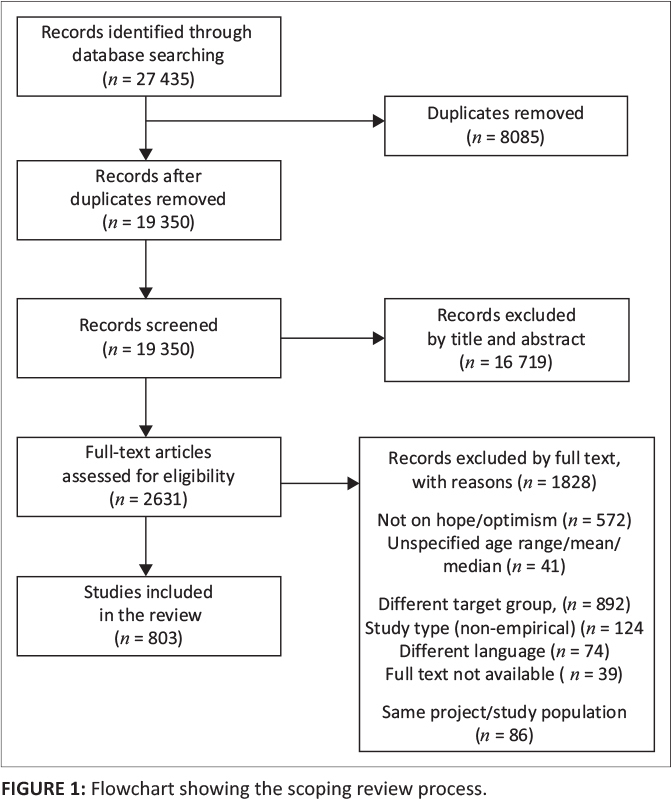
Flowchart showing the scoping review process.

We uploaded the articles identified from the databases search to EppiReviewer Web software (https://eppi.ioe.ac.uk/EPPIReviewer-Web/home). Members of the research team independently screened the study titles, abstracts and full article texts to assess for their eligibility, while A.A. provided guidance. Disagreements between the reviewers, at any stage of the review, were reconciled through discussion and consensus. Table 1 gives a summary of the criteria that was used to select the eligible studies for this review, while Figure 1 shows the selection process of the articles.

**TABLE 1:** Study selection criteria.

Criterion	Inclusion	Exclusion
Geographical location	Global	None
Age	10–19 years (mean/median/age range or relevant grade level)†‡	Age outside 10–19 years
Language	Articles published in English	Articles published in non-English languages
Evidence sources	Empirical studies	Non-empirical studies such as case reports, commentaries, reviews. Book chapters and theses were also excluded

Note: Where there were multiple studies from a similar project, we included the more comprehensive study.

† , For studies that reported only the grade level of the participants without their ages, we included the studies with the reported age grades falling into the 10 to 19-year range based on the available respective country statistics.

‡ , For the studies that included adolescents with children or adults (i.e., the reported age range extending beyond the 10–19 range), we included those studies with the reported mean or median age falling within our target age range.

### Extracting and charting the data

From each of the eligible studies, extracted information included: the name of the author, year of publication, country, adolescent-specific sub-population, age of participants, sample size, measures used, the reported psychometric properties, and the correlates of adolescent optimism or hope. For psychometric properties, we extracted data on reliability (internal consistency, test-retest reliability) and validity data (specifically, criterion, construct, divergent, content, convergent validity, measurement invariance and cross-cultural validity). We also documented translation and adaptation of the tools as reported in the studies.

### Collating, summarising and reporting the results

We calculated the total number of individual measures of hope and optimism that we had identified from the included articles. We then counted the frequency of use of the individual measures identified. In this study, we selected the top 10 most used measures of hope and optimism as our most frequently used measures. Data on reliability and validity of the tools from the selected studies were extracted into Excel and the clean copy was transferred to Microsoft Office Word table. The countries where the data were collected were summarised by continent, and descriptive statistics were used to give the summaries.

### Ethical considerations

Although this study was a scoping review, it was nested under a study aimed at developing measures of adolescent connectedness and character development. For this reason, a research ethics clearance was sought for the entire study by the National Commission for Science, Technology and Innovation (Ethical clearance number: NACOSTI/P/20/6460).

## Results

### Study characteristics

**FIGURE 2:**
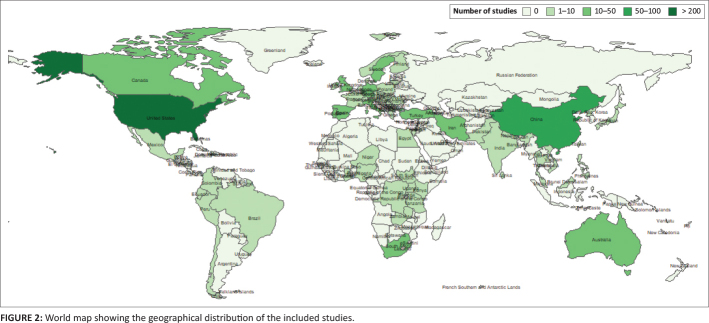
World map showing the geographical distribution of the included studies.

A total of 803 eligible studies were included in this review as shown in the study selection flowchart (Figure 1). Online Appendix 2 provides detailed characteristics of the included studies. The included studies were published from 1976 to 2023, with most of them (*n* = 624, 77.8%) being published from 2010 onwards. Almost half of the included studies (*n* = 341, 42.5%) originated from North America. The remaining studies were distributed across Asia (*n* = 191, 23.8%), Europe (*n* = 179, 22.3%), Oceania (*n* = 32, 4.0%), Africa (*n* = 29, 3.6%) and South America (*n* = 13, 1.6%). A few of the included studies (*n* = 18, 2.2%) were multi-country studies. Figure 2 shows the geographical distribution of the included studies.

We identified a total of 177 measures of optimism, hope or related constructs of positive future orientation used across 751 of the included studies (see Online Appendix 3 for a list of these measures). In the remaining 52 studies, study-specific items were used to assess optimism, hope or related constructs instead of the existing or newly developed measures. This review focuses on measures of optimism and hope. Therefore, in reporting the results, we only focus on measures of optimism and hope. Information on the measures of related constructs of positive future orientation can be found in Online Appendix 2.

### Measures of optimism among adolescents and their psychometric properties

We identified 86 measures of optimism among adolescents used across 360 of the included studies. Online Appendix 6 shows a list of these measures, including their frequency of use. Among these measures, the LOT (Scheier & Carver, 1985), including its revised versions (LOT-R) (Scheier et al., 1994), the YLOT (Ey et al., 2005), the Peer Life Orientation Test (PLOT), including its revised versions (PLOT-R), (Deptula et al., 2006), the Extended Life Orientation Test (ELOT) (Chang et al., 1997), the Engagement, Perseverance, Optimism, Connectedness and Happiness (EPOCH) Measure of Adolescent Wellbeing (Kern et al., 2016), the Positivity Scale (Caprara et al., 2012), the Resiliency Inventory (Song, 2003), the Children’s Attributional Style Questionnaire (Kaslow & Tanenbaum, 1995), the Generalized Expectancy for Success Scale (GESS) (Fibel & Hale, 1978), the Resiliency Scale for Children and Adolescents (RSCA) (Prince-Embury & Prince-Embury, 2007), the Reasons for Living Inventory for Adolescents (RFL-A) (Osman et al., 1998), the Optimism and Pessimism Scale (Levy, 1985), the Connor-Davidson Resilience Scale (CDRS) (Connor & Davidson, 2003), and the Social and Emotional Health Survey–Primary (SEHS-P) (Furlong et al., 2013), were the most frequently used measures. These 14 measures were used across 276 of the included studies assessing optimism. Of these, 230 studies reported an aspect of reliability and/or validity of these measures. We have summarised these psychometric properties in Table 2 and in the Online Appendix 3.

**TABLE 2:** Psychometric properties of the most frequently used measures of optimism among adolescents.

Description	LOT/LOT-R	YLOT	EPOCH	Positivity scale	CASQ	Resiliency inventory	Resiliency scale for children and adolescents	PLOT/PLOT_R	ELOT	RFL-A	Optimism and pessimism scale	GESS	SEHS-P	CDRS
Total number of studies	186	23	19	11	6	5	4	4	3	3	3	3	3	3
Number of studies reporting internal consistency	160	19	15	11	2	5	3	4	3	3	2	3	3	1
Number of studies reporting test-retest reliability	7	1	3	0	0	0	1	1	1	0	0	0	0	0
Total numbers of studies assessing validity	21	4	9	2	0	0	0	1	0	2	0	1	3	0
Number of studies reporting measurement invariance	1	2	9	2	0	0	0	0	0	0	0	1	1	0
Number of studies reporting construct validity	16	4	4	2	0	0	0	1	0	1	0	0	3	0
Number of studies reporting convergent or concurrent validity	6	2	4	1	0	-	0	0	0	2	0	0	1	0
Number of studies reporting divergent validity or discriminant validity	2	1	2	0	0	0	0	0	0	1	0	0	0	0
Number of studies reporting predictive validity	1	1	0	0	0	0	0	0	0	0	0	0	0	0
Number of studies reporting criterion validity	2	0	1	0	0	0	0	0	0	0	0	0	0	0
Number of studies reporting face validity	0	0	1	0	0	0	0	0	0	0	0	0	0	0
Number of studies reporting content validity	0	0	1	0	0	0	0	0	0	0	0	0	0	0

LOT, Life Orientation Test; YLOT, Youth Life Orientation Test; EPOCH, Engagement, Perseverance, Optimism, Connectedness, and Happiness; CASQ, Children’s Attributional Style Questionnaire; PLOT, Peer Life Orientation Test; ELOT, Extended Life Orientation Test; RFL-A, Reasons for Living Inventory for Adolescents; GESS, Generalized Expectancy for Success Scale; SEHS-P, Social and Emotional Health Survey–Primary; CDRS, Connor-Davidson Resilience Scale.

All 230 studies reported the internal consistency of these measures except one study (Saeed et al., 2019) that only reported the test-retest reliability of the EPOCH. Two hundred and twenty-five of these studies reported the internal consistency using Cronbach’s alpha, with the reported alphas above the recommended threshold of 0.70 (Cicchetti, 1994) in all but 61 studies (Online Appendix 2). Of the remaining studies, the internal consistency of these measures was assessed using McDonald’s Omega in four studies (Kennes et al., 2021; Kern et al., 2019; Monzani et al., 2014) and reliability coefficient H in one study (Gaudreau et al., 2015). Twelve of these studies additionally assessed the test-retest reliability of some of these measures (Online Appendix 2). The reported reliability in these studies was above the recommended threshold of 0.40 except in two studies (Kern et al., 2019; Zeng & Kern, 2019).

Forty-two studies assessed the validity of some of these measures, including, measurement invariance, construct, convergent, divergent, discriminant, predictive, criterion, concurrent, content and cross-cultural validity (Online Appendix 2). In establishing cross-cultural validity, only one study (Kern et al., 2019) demonstrated configural (root mean square error of approximation [RMSEA] = 0.042, comparative fit index [CFI] = 0.96, χ^2^ = 2983.5, degree of freedom [*df*] = 480) and weak (RMSEA = 0.045, CFI = 0.95, χ^2^ = 3476.0, *df* = 510) metric measurement invariance of the EPOCH across three countries (Australia, China and the United States).

In 14 of the included studies, only the cross-cultural adaptation of these measures was conducted. However, in most of these studies, only forward translations to the target languages were conducted (Online Appendix 2). Detailed psychometric properties and cross-cultural adaptation and translations of all the measures of optimism are in Online Appendix 2.

### Measures of hope among adolescents and their psychometric properties

Sixty-four measures were used across 414 of the included studies to assess hope in adolescents (see Online Appendix 5 for a list of these measures, including their frequency of use). Among these measures, the CHS (Snyder et al., 1997), the Snyder’s Hope Scale (Snyder et al., 1991), the State Hope Scale (Snyder et al., 1996), the Herth Hope Index (Kaye Herth, 1991), the Hopefulness Scale for Adolescents (HSA) (Hinds & Gattuso, 1991), the Hopefulness about Future Scale (Whitaker et al., 2000), the Trait Hope Scale, the Values in Action Inventory of Strengths for Youth (VIA-Youth) (Park & Peterson, 2006), the Staats’s Hope (Staats, 1989), the Beck Hopelessness Scale (BHS) (Beck et al., 1988), the Hopelessness Scale for Children, and the Work Hope Scale were the most frequently used measures (Table 3). These measures were used across 227 of the included studies assessing hope and their psychometric properties reported in 279 studies. All the studies reported an aspect of reliability of these measures with 62 studies additionally reporting the validity of some of these measures (Table 3 and Online Appendix 2 and Appendix 4).

**TABLE 3:** Psychometric properties of the most frequently used measures of hope among adolescents.

Description	CHS	Snyder’s Hope Scale	HHI	HSA	State Hope Scale	Trait Hope Scale	VIA-Youth	Staat’s Hope Index	BHS	Hopefulness about Future Scale	Hopelessness Scale for Children	Work Hope Scale
Total number of studies	192	55	13	10	11	10	12	7	8	3	3	3
Number of studies reporting internal consistency	161	44	10	9	11	10	10	7	4	3	3	3
Number of studies reporting test-retest reliability	6	2	0	0	0	0	2	0	0	0	0	0
Number of studies reporting inter-rater reliability	0	1	0	0	0	0	0	0	0	0	0	0
Total numbers of studies assessing validity	35	12	3	0	2	1	3	0	0	0	0	0
Measurement invariance	11	1	0	0	1	0	0	0	0	0	0	0
Number of studies reporting construct validity	25	10	3	0	2	1	3	1	0	0	0	0
Number of studies reporting convergent validity/concurrent validity	11	2	2	0	0	0	2	1	0	0	0	0
Number of studies reporting divergent or discriminant validity	3	0	1	0	0	0	0	0	0	0	0	0
Number of studies reporting predictive validity	2	1	0	0	1	0	0	0	0	0	0	0
Number of studies reporting criterion validity	1	1	0	0	0	0	0	0	0	0	0	0
Number of studies reporting face validity	0	0	0	0	0	0	0	0	0	0	0	0
Number of studies reporting content validity	3	0	1	0	1	0	0	0	0	0	0	0

CHS, Children’s Hope Scale; HHI, Herth Hope Index; HSA, Hopefulness Scale for Adolescents; VIA-Youth, Values in Action Inventory of Strengths for Youth; BHS, Beck Hopelessness Scale.

Internal consistency was reported using Cronbach’s alpha in 282 studies and the reported reliability was adequate (Cronbach’s alpha ≥ 0.70) in all but 37 studies (Online Appendix 2). One study (Xiang et al., 2020) reported the internal consistency of the CHS using McDonald’s Omega, and the reported omega was adequate (0.87). Fourteen of the studies additionally reported the test-retest reliability of some of these measures, with the reported reliability above the threshold of 0.40 in all the studies. Interrater reliability was additionally reported in only one study (Cheavens et al., 2019) for the Snyder’s Hope Scale, and was found to be adequate (0.60–0.95).

The types of validity assessed and established across studies for some of these measures include: measurement invariance, construct, convergent, discriminant, incremental, content, criterion, predictive and cross-cultural validity. Cross-cultural validity was reported only for the CHS in only two studies (Savahl et al., 2020; Yang et al., 2021) through measurement invariance (see Table 3 and Online Appendix 4). In these studies, multigroup scalar invariance across English and Afrikaans-speaking South African adolescents (*C* ^2^ = 62.990, *df* = 19, *p* = 0.000, comparative fit index [CFI] = 0.975, root mean square error of approximation [RMSEA] = 0.048, standardised root mean square residual [SRMR] = 0.029) was established (Shazly Savahl et al., 2020), while configural and metric invariance of both the one-factor and two-factor models of the CHS, as well as partial scalar invariance of the one-factor model was demonstrated across Chinese and American cultures (Online Appendix 4) (Yang et al., 2021). In the cross-cultural adaptation of these measures, only 21 studies reported on translations and/or adaptation of measures of hope, most of which were done for the CHS (Online Appendix 2). Detailed psychometric properties, including cross-cultural adaptations and translations of all the measures of hope are in Online Appendix 2.

### General measures of optimism and hope in adolescents

In 13 of the included studies (Afzal et al., 2018; Carmona-Halty et al., 2019; Chen & Kuo, 2020; Ginevra et al., 2018; Santilli et al., 2017, 2019), measures assessing both optimism and hope were used. Four such measures were identified in this review. They include the Visions about Future Scale (Ginevra et al., 2017), the Anila Positive Psychological Capital Scale (APS) (Afzal et al., 2018), the Academic Psychological Capital Questionnaire (Avey et al., 2011), the Inventory of Adolescent Resilience (Chan et al., 2009), the Positive Psychological Capital Questionnaire (PPQ) and My HERO Scale. Among these measures, only the Visions about Future Scale and the PPQ were used in more than one study. The rest of the measures were each used only in one study.

The internal consistency of these measures was reported in all the studies except two studies (Chen & Kuo, 2020; Min & Yao, 2022) that used the Inventory of Adolescent Resilience and the PPQ, respectively. The reported internal consistency for the Visions about Future Scale, the Anila Positive Psychological Capital Scale (APS), the Academic Psychological Capital Questionnaire and the PPQ was adequate (Cronbach’s alpha ≥ 0.70) in all the studies except in only one study (Afzal et al., 2018).

Validity was only assessed for the Visions about Future Scale in three studies (Ginevra et al., 2018; Santilli et al., 2017) and the APS in only one study (Afzal et al., 2018). The types of validity assessed in these studies include measurement invariance across gender, construct, convergent, divergent, discriminant, face, content and concurrent validity. Santilli et al. (2017) examined the cross-cultural validity of the Visions about Future Scale and reported its measurement invariance across Italian and French adolescents (configural: χ^2^ (129) = 451.80, CFI = 0.976, non-normed fit index [NNFI] = 0.970, RMSEA = 0.078; weak: χ^2^ (147) = 490.45, CFI = 0.975, NNFI = 0.972, RMSEA = 0.075; and strong invariance: χ^2^ (129) = 593.25, CFI = 0.969, NNFI = 0.969, RMSEA = 0.079).

## Discussion

We conducted this scoping review to identify the existing measures of adolescent hope and optimism. We specifically aimed to: (1) identify the measures of hope and optimism available for use among adolescents (2) determine the most frequently used measures of adolescent hope and optimism and (3) document the psychometric properties of the identified most frequently used measures of adolescent hope and optimism.

We identified several measures of hope and optimism. The most frequently used measures, among these measures, of optimism were: LOT (Scheier & Carver, 1985), including its revised versions (LOT-R) (Scheier et al., 1994), the YLOT (Ey et al., 2005), the PLOT, including its revised versions (PLOT-R) (Deptula et al., 2006), the (ELOT) (Chang et al., 1997), the EPOCH (Kern et al., 2016), the Positivity Scale (Caprara et al., 2012), the Resiliency Inventory (Song, 2003), the CASQ (Kaslow & Tanenbaum, 1995), the GESS (Fibel & Hale, 1978), the RSCA (Prince-Embury & Prince-Embury, 2007), the RFL-A (Osman et al., 1998), the Optimism and Pessimism Scale (Levy, 1985), the CDRS, and the Social and Emotional Health Survey–Primary (SEHS-P). Notably, LOT/LOT-R is by far the most used measure of optimism in adolescence. On the other hand, the most frequently used measures of hope were: CHS (Snyder et al., 1997), the Snyder’s Hope Scale (Snyder et al., 1991), the State Hope Scale (Snyder et al., 1996), the Herth Hope Index (Kaye Herth, 1991), the Hopefulness Scale for Adolescents (HSA) (Hinds & Gattuso, 1991), the Hopefulness about Future Scale (Whitaker et al., 2000), the Trait Hope Scale, the VIA-Youth (Park & Peterson, 2006), the Staats’s Hope (Staats, 1989), the BHS (Beck et al., 1988), the Hopelessness Scale for Children, and the Work Hope Scale. Of these, the CHS was the most used measure of adolescent hope.

In this review, we identified 154 measures of adolescent hope, optimism or positive future orientation. Most of the included studies were conducted from 2010 onwards. However, there was an unequal geographical distribution of the studies. More than half of the included studies (87.6%) were conducted in North America (particularly the United States), Europe and Asia (particularly China). These are mostly high-income settings (World Bank, 2017). Notably, there were only 27 studies from the African context, yet the region carries the largest proportion of the world’s adolescent population (World Health Organization [WHO], 2014). This highlights the need of more research on adolescent hope and optimism across different settings. Additionally, there were very few studies from Latin America. There are two possible explanations for this observation. Firstly, that this is a true reflection of the scientific literature. Secondly, that it is an artefact of language bias, since we did not look at the manuscripts written in other languages other than English.

Of the studies that reported the psychometric properties of the identified measures, most of the reports were of reliability with a few additionally reporting their validity. Moreover, the studies that reported the psychometric robustness of these measures were also mostly from the Western settings where the measures were developed. For instance, although the LOT/LOT-R was the most commonly used measure of optimism used across 27 different countries, none of these countries was a sub-Saharan African country. Additionally, other measures like the PLOT/PLOT-R and the Hopefulness about Future Scale have only been utilised in the countries where they were initially developed (the United States and China, respectively). This reiterates the need for more validation of these measures, including outside their contexts of origin to determine their cross-cultural validity and stability across different settings. Establishment of cross-cultural validity and utility of measures is salient as it allows for a broader use of tools into low- and middle-income countries, contributes to the expansion of research evidence across contexts, and evaluation of interventions on positive youth development. There is, therefore, a need to invest more in development, adaptation and validation of measures that can be used in low- and middle-income settings where most of the world’s youth reside (WHO, 2014).

We identified 26 measures of hope and optimism that were mostly used across studies. Of these measures, the LOT/LOT-R and the CHS were the most preferred measures of adolescent optimism and hope, respectively. The LOT is a 12-item measure that assesses dispositional optimism (Scheier & Carver, 1985). There is a large body of evidence documenting the reliability and/or validity of this measure (Ayub, 2009; Hale et al., 1992; Terrill et al., 2002). However, subsequent research led to further refinement of the items in the tool and a minor modifications were done to remove two items which were thought to be ‘problematic’ (Scheier et al., 1994). The revised instrument, LOT-R, also showed good psychometric properties (Scheier et al., 1994). These findings were reflected in our review where this measure demonstrated good psychometric properties. Hence, the LOT/LOT-R remains the scale of choice in assessing adolescent optimism. This could be attributed to its brevity, simplicity in administration, and evidence of adequate psychometric properties. However, this evidence is limited in low-to-middle-income settings such as those of Africa. For instance, of the 27 countries in which this tool has been used, only one of them (Egypt) is an African country. Furthermore, only one study has been conducted in this country. This calls for more adaptation and validation of this tool to such settings.

The CHS is a six-item measure of hope developed by Snyder et al. (1997). The initial validation of this tool showed good psychometric properties (Snyder et al., 1997). This tool has since been translated to other languages and used in different settings. In this review, the CHS demonstrated robust reliability and validity across contexts. The reported reliability in most of the included studies ranged from good to excellent. It also seemed to be valid in terms of construct, divergent, convergent, predictive and criterion validity. These results concur with findings of a recent review on the generalisability of the measure (Munoz et al., 2018). As such, researchers largely from United States, China, Australia, and European regions of the world should consider using this tool in assessing hope in adolescents. However, there is still need for further cross-cultural adaptation and validations of the scale in certain regions of the world such as sub-Saharan Africa and Southeast Asia.

Despite the CHS and the LOT/LOT-R being the most dominant measures of hope and optimism, these tools were majorly developed in the 1990s. Since then, new measures of hope and optimism have emerged building on previous work. We identified other frequently used measures. However, there was scanty information on the reliability and/or validity of these measures, including their cross-cultural validations.

We also identified measures assessing both hope and optimism. This could provide further evidence to support the ideology that these two constructs are related as previously mentioned (Maier et al., 2000; Sethi & Seligman, 1994). However, this review also shows that these constructs are distinct as different measures have been used to assess them. Even where one measure was used to assess both constructs, these measures had specific subscales for each construct.

In the included studies, the convergent and divergent validity of measures assessing hope and optimism were primarily evaluated using related instruments. Specifically, commonly employed measures to assess convergent and divergent validity encompassed a wide range of variables such as well-being, depression, anxiety, suicidal thoughts, crime, resilience, connectedness, self-esteem, self-efficacy, internalising and externalising problems, hopelessness and pro-social behaviours, among others (Choi et al., 2021; Lin et al., 2022; Lu, 2022; O’Byrne et al., 2021; Zhao et al., 2021). The findings consistently demonstrated significant positive correlations between measures of hope and optimism and various positively related aspects, suggesting that individuals with higher levels of hope and optimism tend to experience greater well-being, self-esteem, resilience and positive psychological outcomes (Lu, 2022; O’Byrne et al., 2021; Phillips-Salimi et al., 2007; Zhao et al., 2021). Furthermore, these measures exhibited negative correlations with adverse outcomes such as depression, anxiety and stress, reinforcing the notion that hope and optimism act as protective factors against negative psychological states (Piqueras et al., 2019; Scheier & Carver, 1985; Valle et al., 2004).

Generally, in this review we noted that very few studies reported cross-cultural adaptation of the measures. Even when reported, majorly forward and back-translations of the measures were done with very limited cross-cultural adaptations. A vigorous cross-cultural adaptation would evaluate various aspects including semantic, functional and construct equivalence which is not done when we only carry out forward and back-translations. This leaves out other important aspects that ensure cultural adequacy of these measures and could lead to bias (Abubakar & Van De Vijver, 2017; Van de Vijver & Tanzer, 2004). Future studies could ensure a rigorous cross-cultural adaptations and validation of these measures.

### Study strengths and limitations

The major strength of this study lies in the utilisation of a scoping review which captures a broader scope of information. We also included multiple databases and searching from the grey literature which made it rigorous and comprehensive. However, one possible limitation is the inclusion of articles published in only English; hence, papers in non-English languages may have been left out.

## Conclusion

Hope and optimism are important psychological strengths in adolescents with a growing body of knowledge on their respective assessments. There is a wide range of existing measures of these constructs, although the CHS and the LOT/LOT-R appear to be their predominant measures. The existing measures demonstrated sound psychometric properties although this was mostly limited to reliability with scarce evidence on the validity of the tools, particularly cross-cultural validity. There is a need for researchers to further refine and evaluate these tools to ensure optimal use across contexts. Additionally, most of the studies originate from high income settings where these measures were developed. Therefore, there is need for cross-cultural adaptation and validation of the tools to other settings such as low- and middle-income countries.
